# Research on the Angle Measurement Accuracy of Laser Seekers Based on Electrowetting Dual-Liquid Dynamic Zoom Systems

**DOI:** 10.3390/s26175484

**Published:** 2026-08-29

**Authors:** Yingqi Yao, Ru Zheng, Lingyun Wang, Jiayi Qiao

**Affiliations:** 1 College of Optoelectronic Information, Changchun University of Science and Technology, 7089 Weixing Road, Chaoyang District, Changchun 130022, China; 18143006352@163.com (Y.Y.); wlywly8888@126.com (L.W.); 18043736861@163.com (J.Q.); 2 Optoelectronic Precision Instrument Innovation Laboratory, Changchun University of Science and Technology, 7089 Weixing Road, Chaoyang District, Changchun 130022, China

**Keywords:** semi-active guidance seeker, optical design, zoom system, liquid lens, ZEMAX

## Abstract

Traditional zoom semi-active laser seekers cannot adaptively adjust the spot size while maintaining a compact structure, which degrades angle measurement accuracy. This paper proposes a method based on aberration theory to analyze angle measurement designs an electrowetting dual-liquid zoom optical system that meets compactness constraints. The dynamic curvature control architecture replaces traditional mechanical zoom to achieve optimal detection spot for four-quadrant detectors throughout the entire trajectory. The Gaussian bracket method is employed to calculate and distribute the total optical power of the system, and a dynamic zoom optical system meeting compactness requirements is designed and simulated. Quantitative equations relating the spatial position of the liquid lens to aberrations are derived. A global optimization of the liquid lens zoom optical system for the seeker is performed on the ZEMAX 2024 platform, enabling the system to meet the detection requirements of the entire trajectory through voltage control. Design results show a total system length of 61.6 mm, with distortion controlled within 0.1% during continuous focallength adjustment from 30 to 57 mm. A quantitative evaluation model for aberration-induced angle measurement error is established, and calculations indicate a 62.8% reduction in the RMS angle measurement error over the full zoom range.

## 1. Introduction

Laser semi-active guidance technology utilizes a laser designator to emit coded laser radiation, which is reflected by the target and received by the seeker for processing to achieve precise tracking and terminal guidance. It has become a mainstream guidance method due to its high accuracy and strong resistance to interference. The seeker optical system serves as the core signal reception component [1], and its spot quality directly affects hit accuracy [2]. However, traditional fixed-focal-length systems suffer from an inherent contradiction long-range search requires a small spot for energy concentration, while during close-range tracking, spot dispersion can easily lead to detector saturation and angle measurement deviation [3,4].

To address this contradiction, research efforts have turned to dynamic zoom seeker optical systems [5,6,7,8,9]. In 2020, Pu Xiaoqin et al. performed linearity analysis on semi-active laser seeker optical systems and established corresponding relationship between spot position and angle measurement accuracy [10,11,12]. By virtue of their features of having no moving mechanical parts, a compact structure, and rapidly adjustable focal length [13,14,15,16], liquid lenses have accumulated substantial theoretical design and experimental foundations in the field of zoom optical systems [17,18], with demonstrated capabilities for non-mechanical spot array generation, pixel wobulation for resolution enhancement, and beam shaping for uniform illumination, thereby emerging as a versatile platform for dynamic optical systems [19,20]. In 2023, Dong Jian et al. designed a liquid lens zoom optical system for laparoscopes, achieving an MTF greater than 0.3 at 50 lp/mm, validating the application capability of liquid lenses in compact spaces [21,22,23,24,25]. In 2025, Haas et al. demonstrated a liquid-lens-based tunable spot array and wobble system capable of dynamic array adjustment and mechanical-free pixel shifting for improved imaging resolution [26]. Although the issue of detector saturation under large dynamic optical intensity still exists, these works have demonstrated that liquid-lens-based designs can achieve precise spot profile control without mechanical drift [27], laying the foundation for the miniaturization, lightweight design, and high-reliability detection of seekers in complex environments [28,29].

Addressing the bottleneck problem of limited dynamic range of four-quadrant detectors under complex environments leading to decreased angle measurement accuracy, this paper proposes a method for improving angle measurement accuracy based on an electrowetting dual-liquid dynamic zoom system. By designing an optical system with zoom capability, the seeker can adaptively adjust the defocus parameter according to the real-time echo signal intensity, thereby ensuring that the laser spot size remains within the linear response range of the detector throughout the entire projectile attack. This effectively avoids centroid positioning errors caused by spot oversaturation or underexposure, thus ensuring the angle measurement accuracy and stability of the seeker under complex dynamic echo conditions.

## 2. Principle and Performance Parameters of Semi-Active Laser Guidance Head

### 2.1. Operating Principle of Semi-Active Laser Seeker

**Figure 1:**
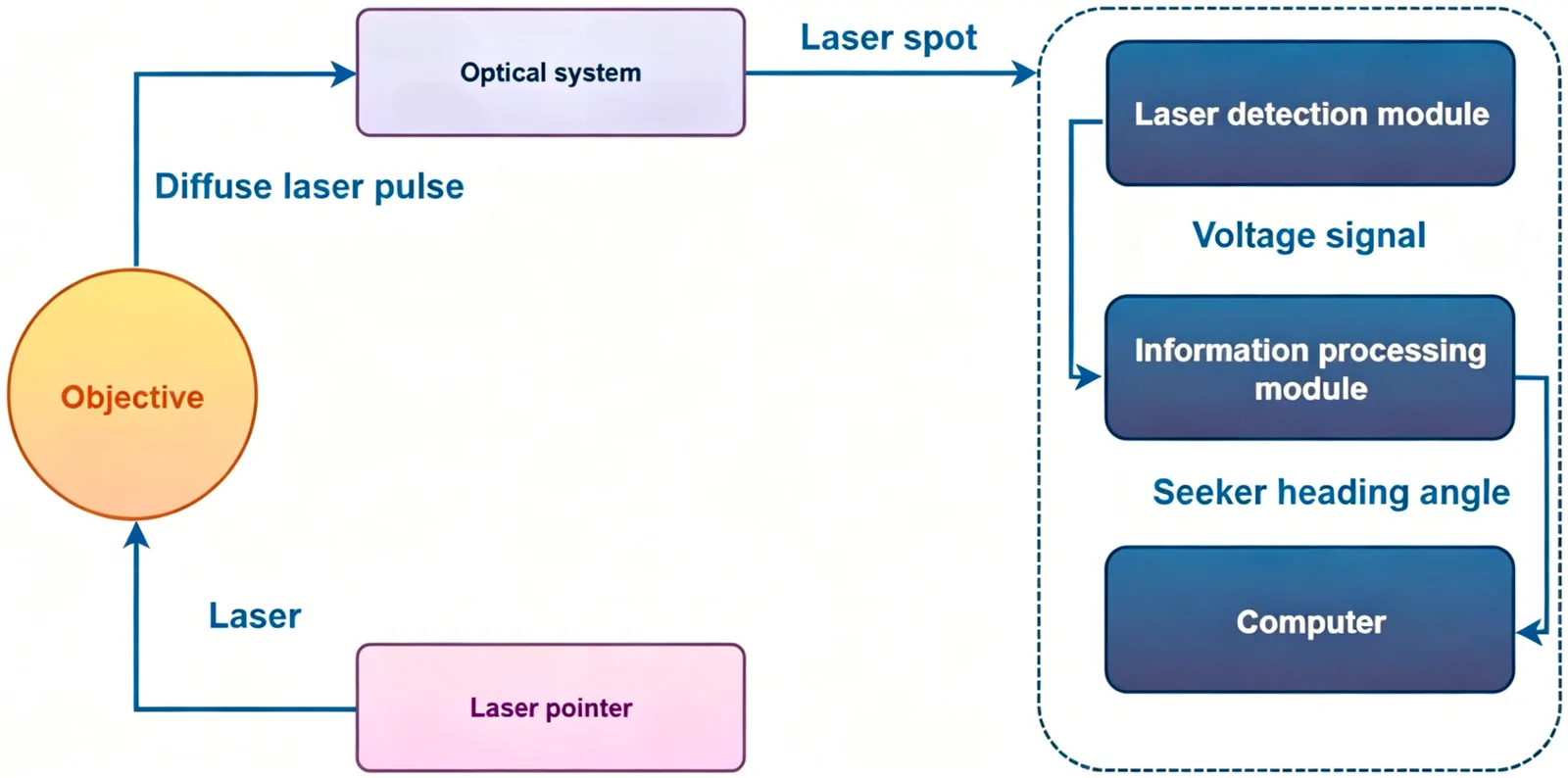
Schematic diagram of semi-active seeker operating principle.

The optical system of a semi-active laser seeker captures the coded laser signal emitted by the external laser designator and reflected by the target in real time, actively focusing and converging it onto the photosensitive surface of the four-quadrant detector. The system calculates and derives the angular deviation parameters of the target relative to the projectile body based on the energy distribution characteristics of the spot received by each quadrant. The control platform then drives the vertical or horizontal fins to deflect for flight attitude correction, achieving precise hits through continuous calibration. The operating principle is shown in Figure 1.

Traditional semi-active laser seekers use mechanical compensation zoom. They achieve focallength variation by moving the zoom and compensation groups axially along guide rails. This makes it difficult to compress the overall optical length, and the system mass typically exceeds 50 g, severely limited by the space and weight constraints. Simultaneously, the reciprocating motion of lens groups causes mechanical clearance and guide rail wear, leading to irreversible optical axis drift. Combined with positioning deviations causing non-reproducible fluctuations in aberration states, the angle measurement accuracy monotonically degrades with increasing zoom cycles and introduces additional random components.

### 2.2. Design Specifications of Semi-Active Laser Seeker

The semi-active laser seeker operates at a wavelength of 1.064 μm, with a receiving aperture of 15 mm, and requires distortion ≤1% within a field of view of ±4°. Detailed design parameters are shown in Table 1.

## 3. Design of Mechanical Compensation and Electrowetting Zoom Optical Systems

The Gaussian bracket method is employed to accurately calculate and distribute the total optical power of the system, thereby determining the initial structure of the zoom optical system. Based on this, a mechanical compensation zoom system and a dual-liquid zoom system based on the electrowetting effect are designed respectively: the former serves as a performance benchmark for the conventional approach, while the latter achieves mechanical-motion-free zooming by adjusting the liquid curvature through voltage, verifying its feasibility in principle.

### 3.1. Initial Structure Design

**Figure 2:**
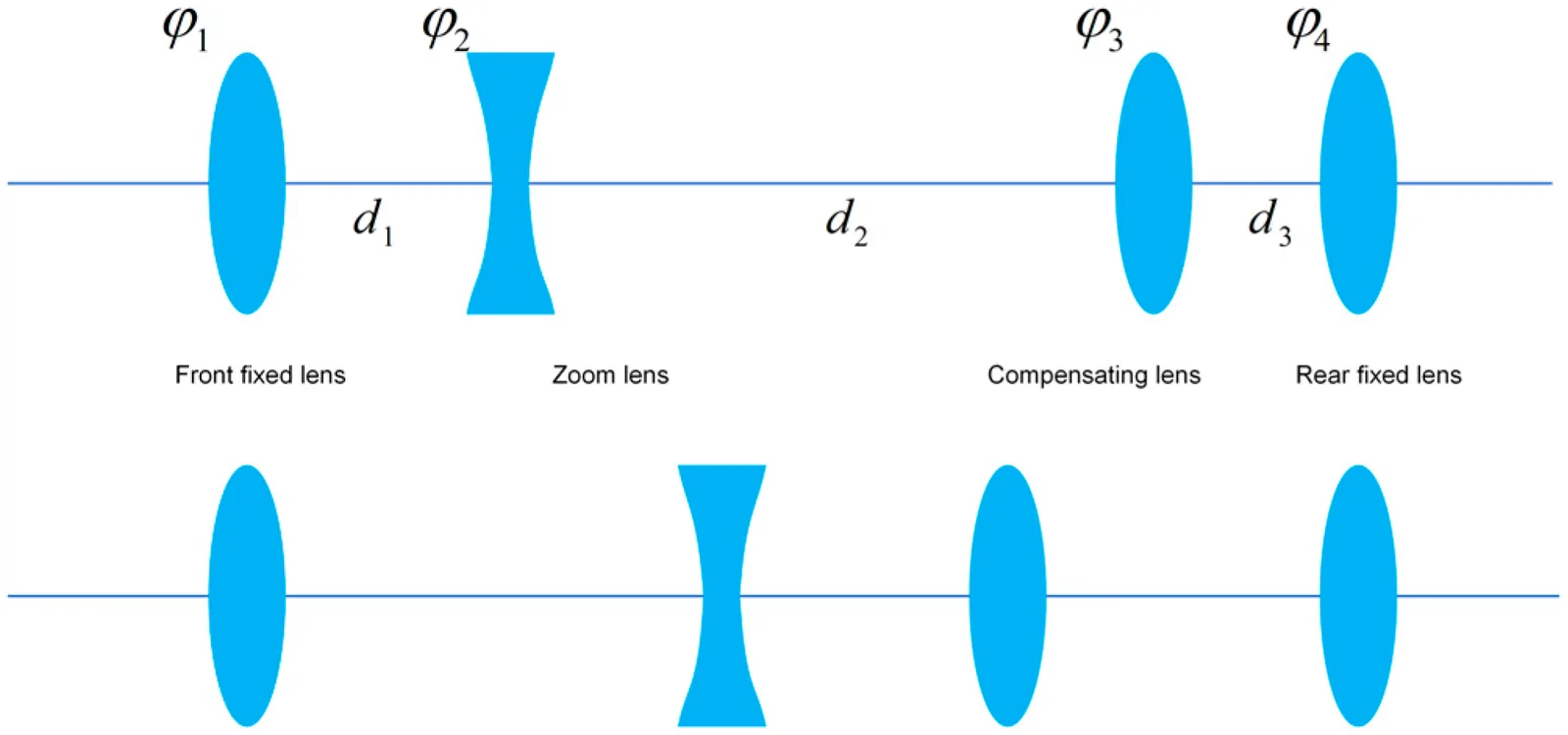
Schematic diagram of a typical traditional zoom optical system.

Traditional zoom optical systems require constant conjugate distance and stable imaging quality during focallength or magnification variation. The basic structure is shown in Figure 2, where the compensation group performs corresponding compensation adjustment according to the displacement of the zoom group.The blue line in Figure 2 represents the chief ray (principal ray) path through the system, showing the imaging relationship during zooming.

Before proceeding with the optical system design, the optical power must be determined using the Gaussian bracket method. The Gaussian bracket method expresses Gaussian constants in a specific bracket form, providing a systematic method for deriving the optical power matrix of paraxial optical systems. Using Gaussian brackets to represent Gaussian constants:

(1) $$A_{j}=\left[\varphi_{i}-d_{i},\varphi_{i+1},-d_{i+1},\ldots ,\varphi_{j-1},-d_{j-1}\right]$$

(2) $$B_{j}=\left[-d_{i},\varphi_{i+1},-d_{i+1},\ldots ,\varphi_{j-1},-d_{j-1}\right]$$

where A, B, C, D are Gaussian constants described by Gaussian brackets, $\varphi_{i}$ is the optical power of the i-th lens group, and $d_{i}$ is the optical interval between the i and (i + 1) lens groups. The recurrence rule for Gaussian brackets is

(3) $$\left[a_{1},a_{2},\ldots ,a_{n}]=[a_{1},a_{2},\ldots ,a_{n-1}]a_{n}+[a_{1}+a_{2},\ldots ,a_{n-2}\right]$$

Therefore, the initial structure of the three-group zoom system shown in Figure 2 can be expressed using Gaussian brackets as

(4) $$\Phi =\left[\varphi_{1},-d_{1},\varphi_{2},-d_{2},\varphi_{3}\right]$$

(5) $$D=d_{1}+d_{2}+blf$$

where Φ is the total optical power of the system, D is the total system length, and blf is the distance from the vertex of the last optical element surface to the focal plane. Further simplification using the properties of Gaussian brackets gives:

(6) $$\left\{\begin{array}{l} \phi =C_{1}\varphi_{3}+C_{2}\\ C_{1}=\left[\varphi_{1},-d_{1},\varphi_{2},-d_{2}\right]=-d_{2}\left[\varphi_{1},-d_{1},\varphi_{2}\right]+\left[\varphi_{1},-d_{1}\right]\\ C_{2}=\left[\varphi_{1},-d_{1},\varphi_{2}\right]=\varphi_{2}\left[\varphi_{1},-d_{1}\right]+\varphi_{1} \end{array}\right.$$

From this, the functional relationship between the optical powers of the zoom and compensation groups and other system parameters can be derived:

(7) $$\varphi_{2}=\Phi -\frac{\varphi_{3}}{E_{2}}-\frac{\varphi_{1}}{E_{1}}$$

In the formula $E_{1}=[\varphi_{1},-d_{1}],E_{2}=[1-d_{2},\varphi_{3}]$.

### 3.2. Mechanical Compensation Zoom System Design

According to the seeker design specifications given in Table 1 and the target zoom range of 30 mm to 60 mm, the optical power distribution is first performed using the Gaussian bracket method. The system is simplified to a three-group structure comprising a zoom group, compensation group, and rear fixed group. Let the optical powers of the groups be φ_2_, φ_3_, φ_4_ respectively, and the air spacings between groups be d_12_, d_23_, d_34_. The total system optical power φ and total length L satisfy Equation (6). By solving the Gaussian equations for the short focal and long focal extreme positions simultaneously, the initial solution for the three-group optical powers is obtained: φ_2_ = 0.018 mm^−1^, φ_3_ = 0.012 mm^−1^, φ_4_ = 0.025 mm^−1^, corresponding to focal lengths of 55.6 mm, 83.3 mm, and 40 mm respectively.

Based on this, an initial structure is built in ZEMAX. Common optical glass materials are selected for the lenses. The radii of curvature are back-calculated from the thin lens power formula $\rho =\Delta n(\frac{1}{r_{1}}-\frac{1}{r_{2}})$. Lens thicknesses are set to a minimum process thickness of 2.5 mm. A multi-configuration setup is used to define six equally spaced focallength positions, with the displacements of the zoom and compensation groups set as variables. The merit function uses RMS spot radius combined with custom operands to control distortion ≤1% and total system length ≤70 mm. The optimization algorithm first employs global optimization to search for an initial solution, then switches to the damped least squares method for local convergence.

**Figure 3:**
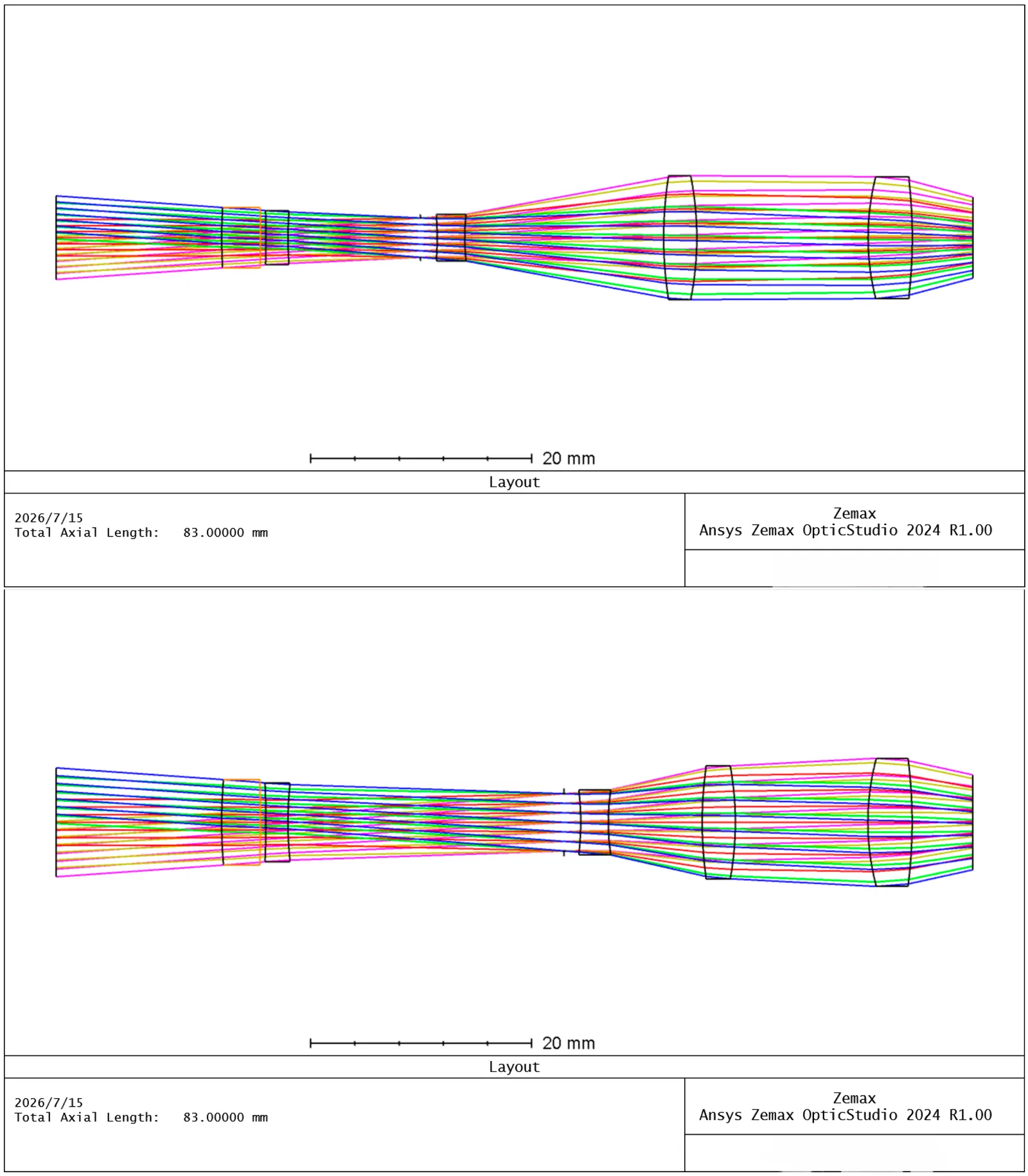
Optimized mechanical compensation zoom optical system.

The optimized system structure is shown in Figure 3. The total system length is 68 mm, and the axial movement strokes of the two zoom lens groups are 4.5 mm and 2.8 mm respectively. At the short focal end, the full-field RMS spot radius is 3.139 mm; at the long focal end, it is 2.283 mm, both smaller than the typical pixel size of a four-quadrant detector, meeting energy concentration requirements. The maximum distortion over the full zoom range is 0.517%, below the 1% design constraint. The maximum separation between tangential and sagittal field curvatures is 0.05 mm, indicating an acceptable level of astigmatism.

### 3.3. Liquid Lens Optical System Design

#### 3.3.1. Liquid Lens Design

**Figure 4:**
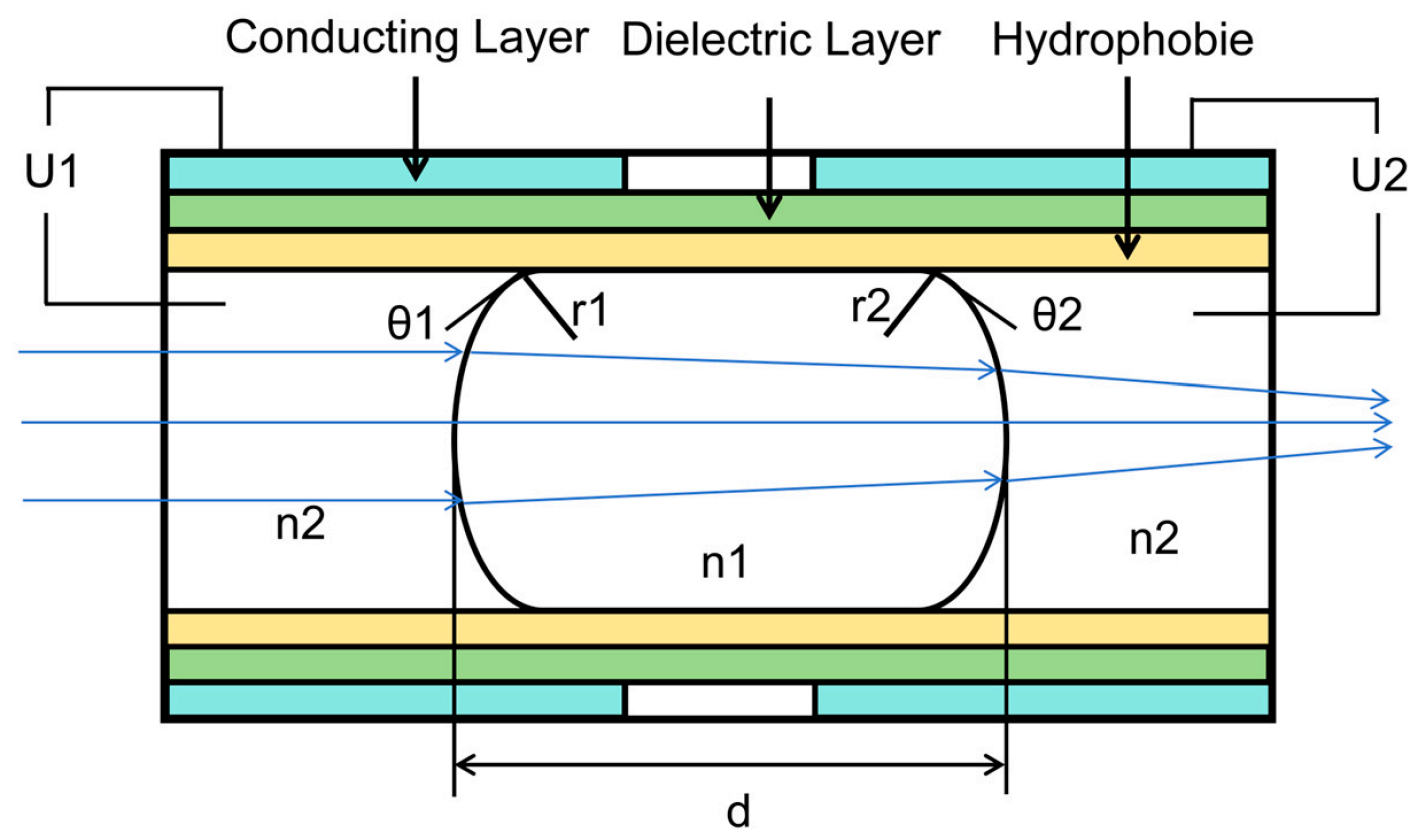
Schematic structure of the dual-liquid lens.

This study selects a dual-liquid lens whose liquid liquid interface curvature can be controlled by voltage as the core zoom element. Its container inner wall is coated with conductive, dielectric, and hydrophobic layers, and a segmented electrode design is employed for independent voltage control. The electrowetting effect changes the contact angle between the conductive liquid and the hydrophobic layer, thereby continuously adjusting the lens focal length, offering advantages of compact structure and high control precision. Figure 4 shows the schematic structure of the dual-liquid, the arrow points in the direction of light.

The focal length of the electrowetting liquid lens is related to the applied voltage. The physical mechanism of the electrowetting effect can be summarized by the equation:

(8) $$\cos\theta =\cos\theta_{0}+\frac{\varepsilon_{r}\varepsilon_{0}}{2d\tau_{12}}U^{2}$$

where θ_0_ is the initial contact angle, *ε* is the relative permittivity of the dielectric layer, $\varepsilon_{0}$ is the vacuum permittivity, $\tau_{12}$ is the interfacial tension.

According to Gaussian optics theory, the relationship between the optical power and curvature of the liquid lens shown in Figure 4 is

(9) $$\frac{1}{f}=\frac{\left(n_{1}-n_{2})(n_{1}r_{1}+(n_{2}-n_{1})\left(\frac{n_{1}r_{1}}{\left(n_{1}-n_{2}\right)}-d\right)\right)}{n_{0}n_{1}r_{1}r_{2}}$$

**Figure 5:**
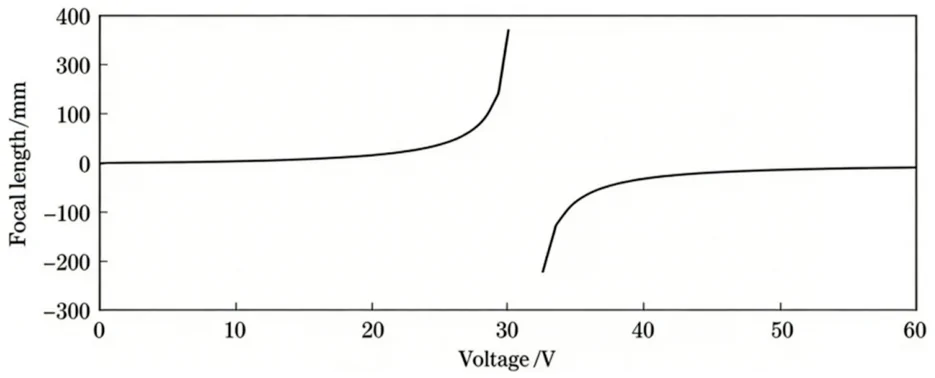
Simulated voltage–focallength curve using MATLAB.

In the formula, $r_{1}=-\frac{r_{0}}{\cos\theta_{1}},r_{2}=-\frac{r_{0}}{\cos\theta_{2}}$, where $r_{0}$ is the half-diameter of the cylindrical container, θ_1_ and θ_2_ represent the contact angles between the two liquid interfaces and the wall, $n_{0}$ is the refractive index of air, $n_{1}$ and $n_{2}$ are the refractive indices of the insulating liquid and conductive liquid respectively, $U_{1}$ and $U_{2}$ are the voltages applied between the conductive liquid and the hydrophobic dielectric layer, $r_{1}$ and $r_{2}$ are the radii of curvature of the two spherical liquid interfaces, and d is the distance between the two liquid interfaces. From the equation, it can be seen that changing the voltage changes the focal length of the liquid lens. Figure 5 shows the voltage–focal length relationship. This relationship was obtained by numerical simulation in MATLAB R2020b. The simulation is based on Equations (7) and (8). It incorporates the electrowetting curve. That curve is derived from the Lippmann Young equation. The simulation also assumes a linear dielectric layer response. This assumption holds within the operating voltage range.

#### 3.3.2. Liquid Lens Zoom Optical System Design

This paper employs electrowetting liquid lenses to replace the zoom group and compensation group of the zoom system shown in Figure 3, achieving the goal of focusing without mechanical structures. ZEMAX software is used to simulate the required electrowetting liquid lenses by representing them as lenses with different focal lengths.

The electrowetting dual-liquid lens is modeled in ZEMAX as a two-interface component. The first interface separates air and the insulating liquid with refractive index n_1_ = 1.55 (at 1.064 μm). The second interface separates the insulating liquid and the conductive liquid with refractive index n_2_ = 1.35. The curvature of the liquid liquid interface is set as a variable in each multi-configuration position, while the other interface curvatures and the container glass windows are fixed. The clear aperture is set to 15 mm to match the entrance pupil diameter. A spherical surface assumption is adopted for the liquid liquid interface, neglecting non-spherical effects from gravity or container wall wetting. This simplification may underestimate higher-order aberrations but is consistent with the paraxial initial design stage.

To further optimize system performance, a quantitative comparison of the relationship between liquid lens spatial position and aberration is conducted. A multi-configuration model is established to evaluate the aberration characteristics of three schemes during the zoom process. From aberration theory, the total system aberration is the sum of the contributions of each lens element. Changing the optical power of the liquid lens introduces additional aberration variations. For quantitative evaluation, the sensitivity coefficient S of the system wavefront aberration to the liquid lens optical power is calculated, defined as the ratio of the change in system wavefront aberration to the change in liquid lens optical power:

(10) $$S=\frac{\partial W}{\partial \phi }$$

where W is the system wavefront aberration and ϕ is the optical power of the liquid lens. The calculation indicates that for the position of the second lens element, the sensitivity coefficient is $S_{1}\approx 0.13\lambda$; for the third lens element position $S_{2}\approx 0.15\lambda$; and for the last lens element position $S_{3}\approx 0.08\lambda$. Since ${S_{3}<S}_{1}<S_{2}$, it demonstrates that when the last lens element is the liquid lens, the system aberration is less sensitive to changes in its optical power.

Additionally, the influence of optical power distribution on aberration is considered. The total system optical power is the sum of the optical powers of each element. For a thin lens system, the total optical power can be approximated as

(11) $$\phi_{\mathrm{total}}=\sum \phi_{i}-\sum_{i≺j}d_{ij}\phi_{i}\phi_{j}$$

where $d_{ij}$ is the distance between the lenses. Therefore, the aberration variation caused by a change in liquid lens optical power is approximately proportional to the liquid lens power itself:

(12) $$\Delta W\propto \Delta \phi \cdot \phi_{i}$$

In the optimized system, the last lens element has a relatively small optical power. Therefore, for the same amount of optical power adjustment, the liquid lens located at the last position induces the smallest aberration disturbance among the three candidate schemes.

Combining the sensitivity coefficient analysis and optical power distribution considerations, placing the liquid lens as the last element in the system is the optimal configuration. This configuration minimizes the drift and size fluctuation of the spot during zooming, providing higher control precision and system stability for applications requiring a stable output spot.

**Figure 6:**
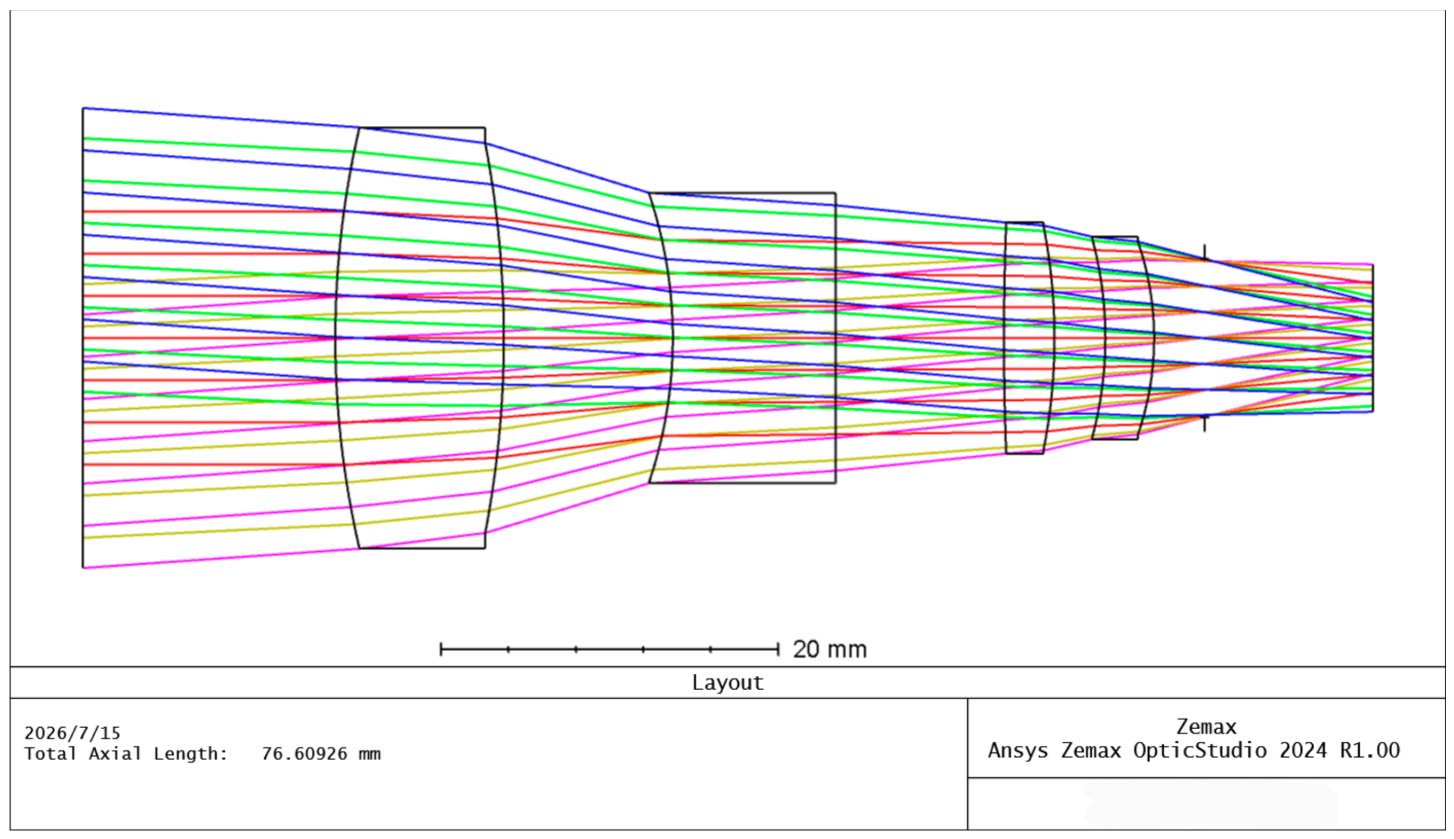

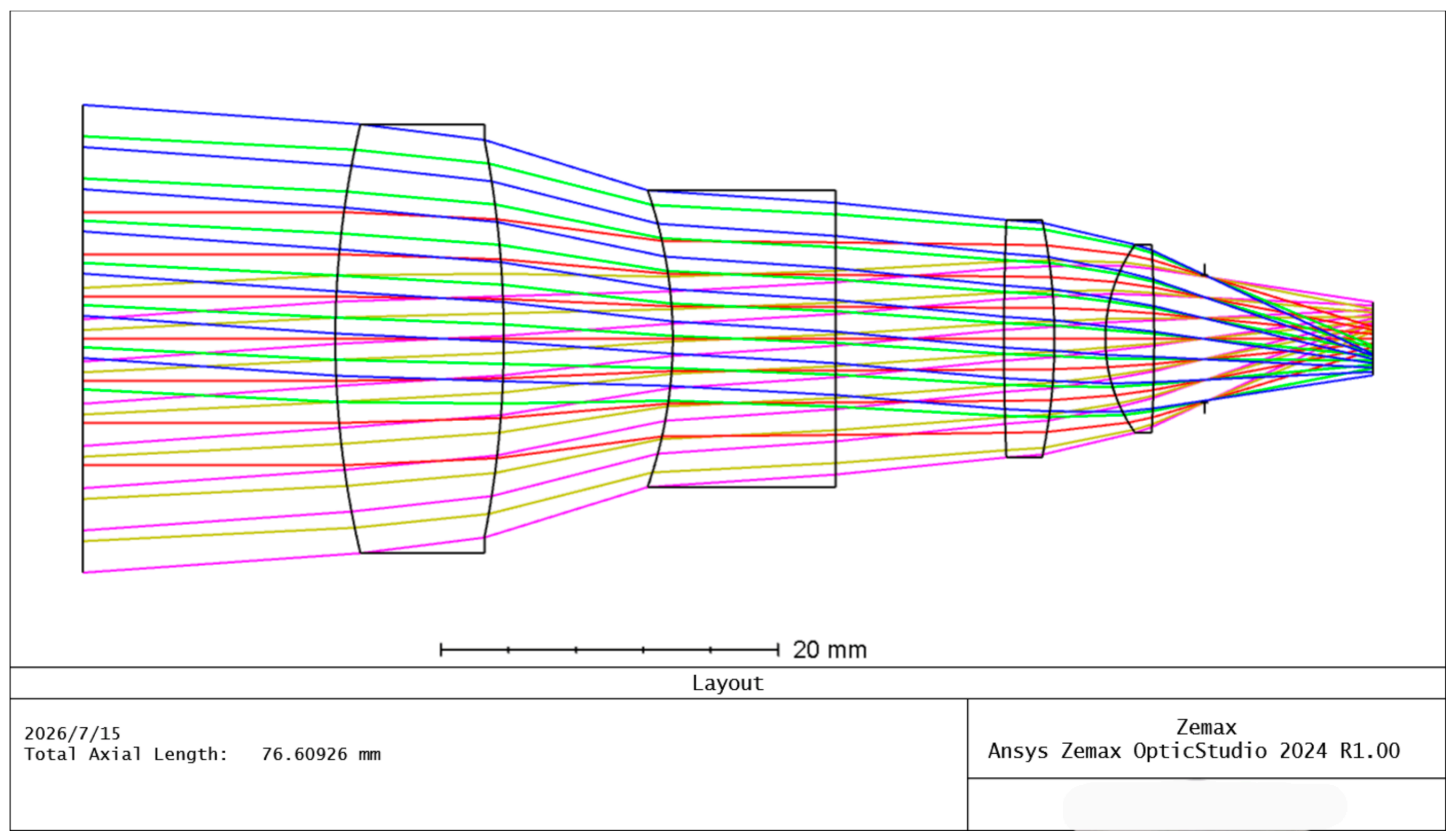
Optimized liquid lens zoom optical system.

Since the liquid lens itself does not have independent aberration correction capability, compensation must be achieved through structural optimization. This involves reasonably distributing optical powers to control spherical aberration and ensure beam convergence on the lens surface, adjusting the aperture stop position to suppress distortion and improve coma, and optimizing the protective cover glass surface to assist in correcting chromatic aberration. Finally, multi-configuration setup and optimization constraints ensure imaging quality. The optimized initial structure of the liquid lens zoom system is shown in Figure 6. In Figure 6, the liquid lens corresponds to the last lens element at the rightmost position. Rays are shown propagating from left to right, clearly extending to the image plane.

### 3.4. Image Quality Evaluation and Performance Analysis

#### 3.4.1. Evaluation of Mechanical Compensation Optical System

**Figure 7:**
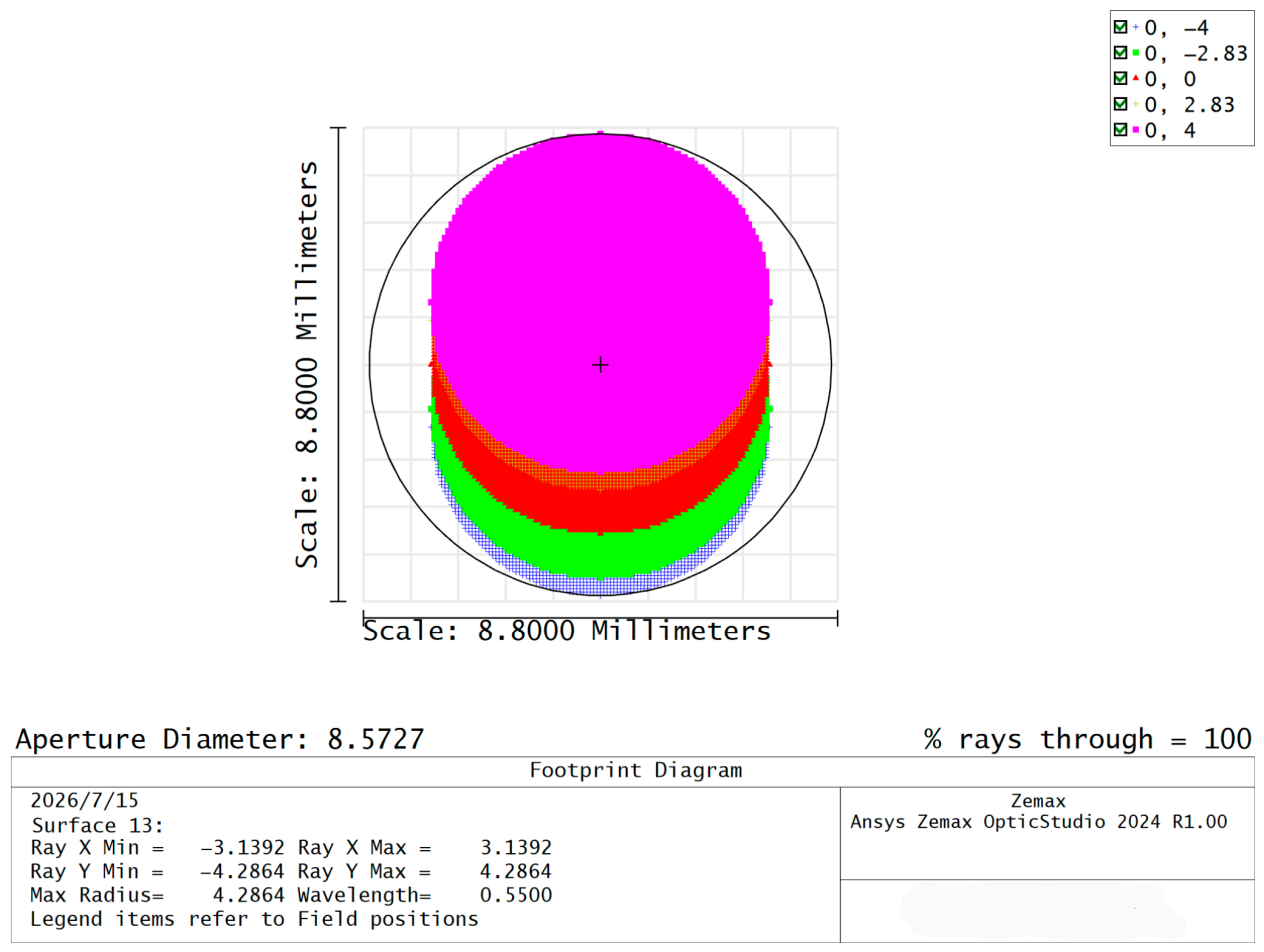

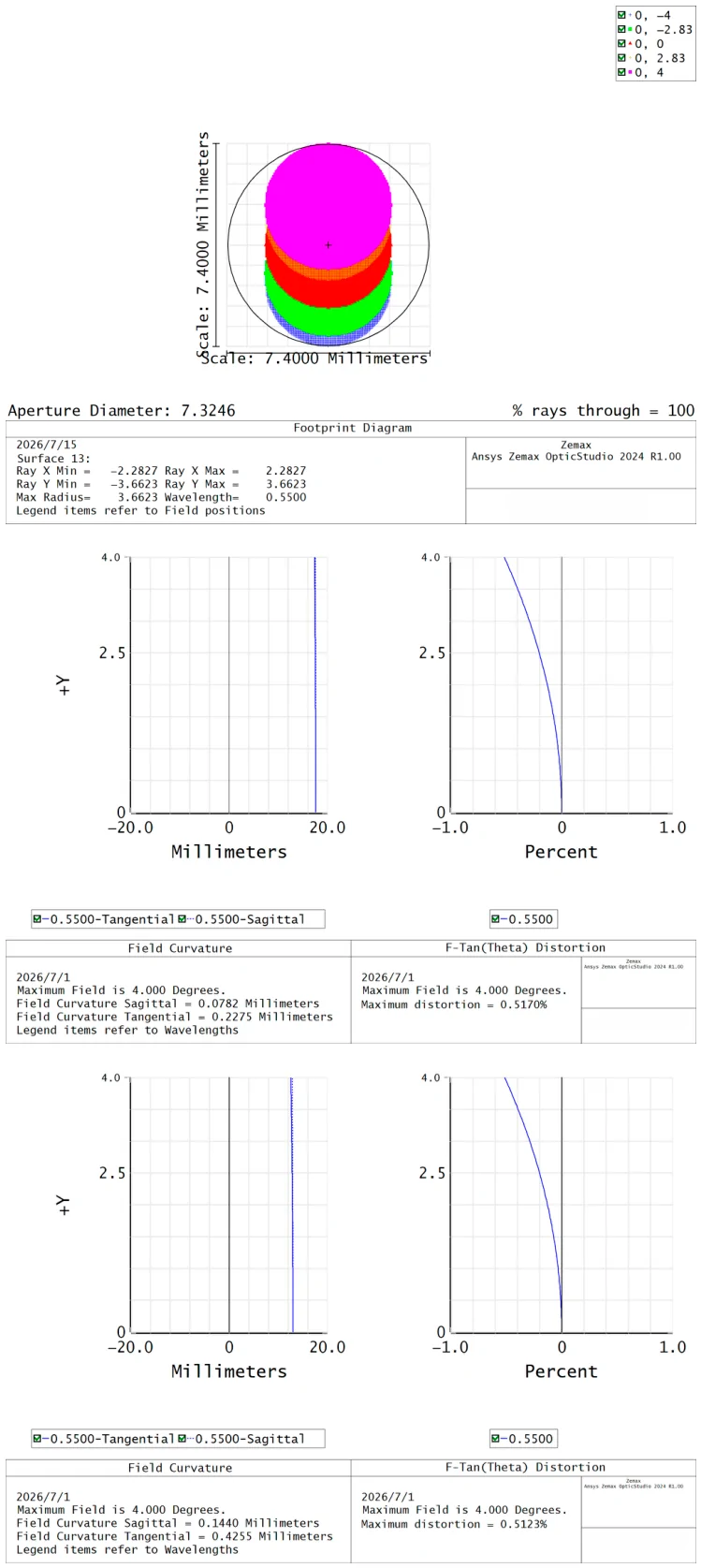
Light trace image and distortion curve of a mechanically compensated zoom optical system.

The imaging performance of the optimized mechanical compensation zoom system at the extreme positions is evaluated using the light trace image and field curvature/distortion curves shown in Figure 7. Regarding spot size, the spot radius is 3.139 mm at the short focal end and 2.283 mm at the long focal end, both smaller than the photosensitive surface radius, satisfying the energy concentration requirement. Field curvature analysis shows that the maximum separation between tangential and sagittal field curves over the full field of view remains minimal at both focallength extremes, indicating negligible difference between the two focal surface positions and good focusing consistency for off-axis image points. Regarding distortion, the maximum relative distortion is 0.517% at the short focal end and 0.512% at the long focal end, both below the 1% design tolerance, indicating acceptable impact on image height linearity. The ray tracing diagram indicates that the distortion of the mechanically compensated zoom system and the focusing consistency of off-axis image points are well-controlled, although the zoom range is slightly insufficient.

In terms of structural compactness, the optimized scheme has an optical total length of 68 mm and an estimated total mass of approximately 55 g, providing a viable baseline for layout under projectile-borne environment constraints.

#### 3.4.2. Evaluation of Dual-Liquid Zoom Optical System

**Figure 8:**
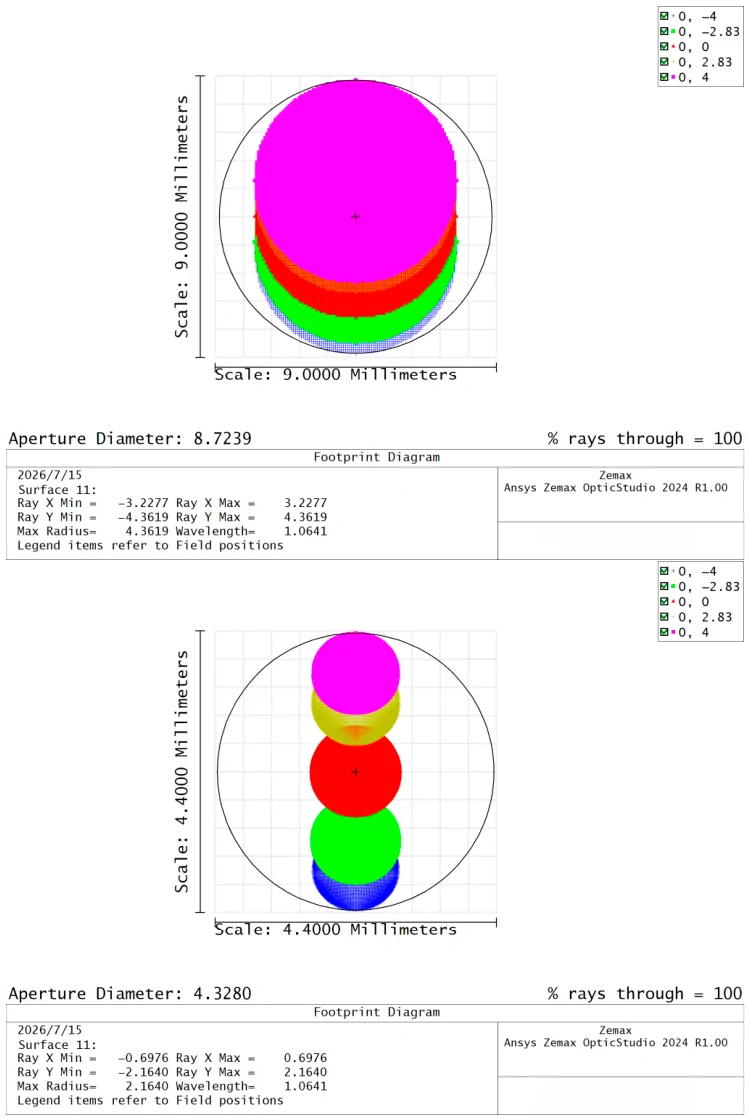

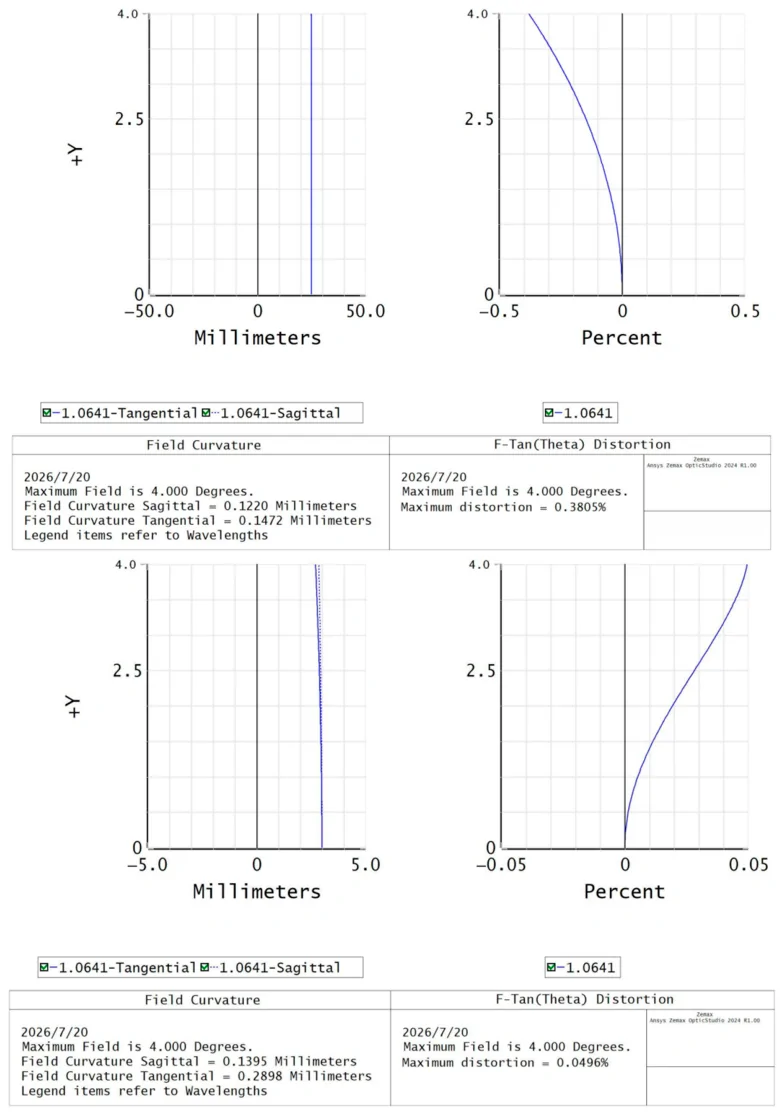
Light trace image and distortion curve of the dual-fluid zoom optical system.

The imaging performance of the optimized dual-liquid zoom system is evaluated using the light trace image and field curvature/distortion curves shown in Figure 8. Regarding spot size, the spot radius is 3.228 mm at the short focal end and 0.698 mm at the long focal end, both smaller than the photosensitive surface radius, satisfying the energy concentration requirement. The spot size at the long focal end is reduced by approximately 69% compared to the conventional system, indicating significantly improved energy concentration. The field curvature curves show that the maximum separation between tangential and sagittal fields at both focal ends is significantly smaller than that of the conventional system, demonstrating superior focusing quality for off-axis image points. Regarding distortion, the maximum relative distortion is 0.038% at the short focal end and 0.049% at the long focal end, demonstrating excellent image height linearity. Compared with the mechanically compensated zoom system, the zoom range is significantly increased.

In terms of structural compactness, the optical total length of this scheme is 61.6 mm, and the total mass is approximately 17.9 g. Compared to the conventional system, the axial dimension is reduced by 9.4% and the mass is reduced by 67.5%. The dual-liquid lens eliminates mechanical moving parts, fundamentally avoiding issues such as optical axis drift, wear, and non-reproducible aberration fluctuations.

Based on the comprehensive evaluation above, the optimized dual-liquid zoom optical system outperforms the mechanical compensation system in both image quality and structural compactness. More importantly, the dual-liquid system achieves zooming without moving parts, fundamentally avoiding the image quality degradation and reliability risks associated with mechanical wear and clearance in conventional systems. While maintaining equivalent or superior image quality, its axial length is reduced by 9.4% and mass by 67.5%, making it better suited to the stringent requirements of projectile-borne seekers for miniaturization and high reliability.

## 4. Analysis of Seeker Angle Measurement Error

### 4.1. Influence of Aberrations on Angle Measurement Accuracy

All analyses in this section are based on simulated data from the ZEMAX optical design software, not on physical prototypes. The angle measurement principle of the four-quadrant detector is based on the small-angle approximation:

(13) $$\theta \approx \frac{\delta }{f}$$

where δ is the spot displacement on the image plane, and f is the system focallength. Differentiating this equation and ignoring focallength calibration errors and non-optical factors, the angle measurement error due to aberrations is

(14) $$d\theta_{ab}=\frac{\delta_{ab}}{f}$$

where $\delta_{ab}$ is the centroid offset of the spot energy caused by the combined action of various residual aberrations. For semi-active laser seeker optical systems, the contributions of different aberrations to the angle measurement error are shown in Table 2.

From Table 2, the directly contributing aberrations are coma, astigmatism, and distortion, among which coma and astigmatism are asymmetric aberrations that constitute the main sources of angle measurement error and cannot be completely eliminated by calibration.

In ZEMAX, the centroid offset is computed for each focal length and field of view. The operand CENY returns the Y-coordinate of the centroid of the geometric spot; the operand PARY returns the Y-coordinate of the paraxial chief ray, which serves as the reference. The centroid offset $\delta_{ab}$ is then defined as their difference.

(15) $$\delta_{ab}=|\mathrm{CENY}-\mathrm{PARY}|$$

### 4.2. Comparison of Angle Measurement Errors over Full Zoom Range

To systematically evaluate the variation angle measurement error during the zoom process, data were extracted at five equally spaced focallength positions within the range of 30 to 60 mm. Due to the limited tuning range of the liquid lens, the selected focal lengths were 30, 36, 42, 48, 54, and 57 mm.

**Figure 9:**
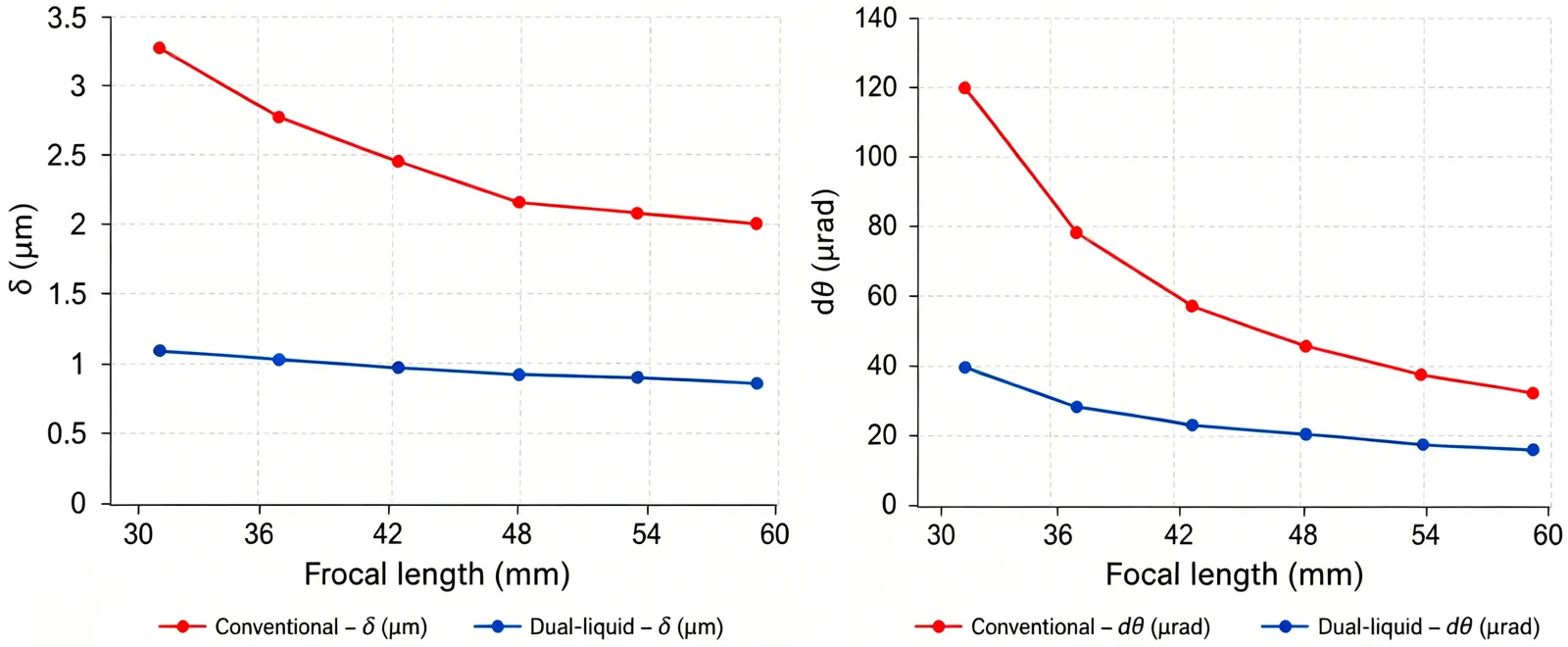
Comparison of centroid offset and angle measurement error as functions of focal length between the two optical systems.

To visually demonstrate the performance difference between the two systems at different focal lengths, line charts are plotted based on the data in Table 3 and Table 4, as shown in Figure 9. The left chart shows the centroid offset as a function of focal length, and the right chart shows the angle measurement error as a function of focal length.

From Figure 9, it can be seen that both the centroid offset and angle measurement error of the two systems decrease with increasing focal length. The dual-liquid system exhibits lower values at all focal lengths compared to the conventional system. The difference is most significant at the short focal end and gradually diminishes as the focal length increases.

Using the full field of 4.0° as a reference, the RMS angle measurement error over the full zoom range is calculated.

(16) $$d\theta_{\mathrm{RMS}}=\sqrt{\frac{1}{N}\sum_{i=1}^{N}{\left(d\theta_{i}\right)}^{2}}$$

Table 5 compares the angle measurement error of the two systems over the full zoom range. At the short focal end, the dual-liquid-lens system achieves 36.88 μrad versus 119.63 μrad for the conventional system; at the long focal end, the values are 16.07 μrad and 36.11 μrad, respectively. This demonstrates that the dual-liquid-lens system provides significantly higher angular measurement accuracy across the entire zoom range.

The reduction ratio is

(17) $$\eta =\left(1-\frac{25.04}{69.68}\right)\times 100\%=64.1\%$$

This value characterizes the reduction the angle measurement error component directly introduced by aberrations in the dual-liquid system compared to the conventional system over the full zoom range.

In summary, the advantage of the dual-liquid system is particularly prominent at the short focal end, while it is comparable to the conventional system at the long focal end. If the characteristic of no mechanical moving parts in the dual-liquid system is further considered, its actual accuracy advantage in full-trajectory dynamic zoom scenarios would be significantly greater than the static comparison values shown above.

## 5. Conclusions

This paper completes the design and simulation analysis of a dynamic zoom optical system for semi-active laser seekers, using an electrowetting-driven dual-liquid lens as the core zoom element. The corresponding control relationship between the driving voltage and the system focallength range is established, achieving continuous zoom from a short focal length of 30 mm to a long focal length of 57 mm. Image quality evaluation results show that the full-field RMS spot radii are 3.228 mm at the short focal end and 0.698 mm at the long focal end, both smaller than the typical pixel size of a four-quadrant detector, indicating that spot energy concentration meets the requirements for centroid extraction accuracy. The maximum relative distortion is 0.07% at the short focal end and 0.034% at the long focal end, maintaining excellent low-distortion characteristics over the full zoom range, which can effectively suppress angular position calculation errors introduced by image plane deformation. The total optical length of the system is 61.6 mm, and the total mass is approximately 17.9 g. Compared to the conventional mechanical compensation zoom scheme, the axial dimension is reduced by 9.4% and the mass is reduced by 67.5%. The zoom process is achieved by adjusting the curvature of the dual-liquid interface through voltage, with the image plane position remaining constant during focallength variation. No mechanical moving parts are involved, providing inherent advantages in response speed and structural reliability. The results are consistent with recent advances in non-mechanical beam shaping [26,27,28], confirming the viability of electrowetting lenses for compact seeker systems, offering an effective technical approach for improving the compactness and full-trajectory angle measurement consistency of projectile-borne optical systems. In practical projectile-borne applications, thermal effects can alter the refractive indices and interfacial tension of the two liquids, causing focallength drift. The liquid liquid interface is also susceptible to laser-induced damage under high-power pulsed irradiation, as reported in similar electrowetting systems [28].

## Figures and Tables

**Table 1:** Technical Parameters of the Semi-Active Laser Seeker.

Parameter	Value
Operating wavelength	1.064 μm
Photosensitive surface diameter	Φ11.4 mm
Field of view	±4°
Entrance pupil diameter	15 mm
Spot uniformity	≥95%
Distortion	≤1%

**Table 2:** Contribution mechanism of various aberrations to angle measurement error.

Aberration Type	Symmetry	Effect on Centroid Shift	Effect on Angle Measurement Error
Spherical Aberration	Rotational symmetry	No centroid shift	Only broadens the spot, indirectly reduces centroid positioning SNR
Coma	Asymmetric	Centroid shift increases linearly with field of view	Primary error source, not correctable by calibration
Astigmatism	Bilateral symmetry about tangential/sagittal planes	Centroid shift increases linearly with field of view	Secondary error source, not correctable by calibration
Field Curvature	Rotational symmetry	No centroid shift	Defocus causes spot broadening, indirectly affects accuracy
Distortion	Rotational symmetry	Image height deviates from ideal value	Can be compensated by calibration

**Table 3:** Angle measurement error at each focal length for the conventional zoom system.

Focal Length (mm)	$\delta_{ab}$ (μm)	$d\theta_{ab}$ (μrad)
30	3.20	119.63
36	2.85	79.17
42	2.50	59.52
48	2.25	46.88
54	2.10	38.89
57	2.05	36.11

**Table 4:** Angle measurement error at each focal length for the dual-liquid zoom system.

Focal Length (mm)	$\delta_{ab}$ (μm)	$d\theta_{ab}$ (μrad)
30	1.08	36.88
36	1.05	29.17
42	1.00	23.81
48	0.98	20.42
54	0.95	17.59
57	0.92	16.07

**Table 5:** Comprehensive comparison of angle measurement error over full zoom range for the two systems.

Parameter (μrad)	Conventional Zoom Optical System	Dual-Liquid Lens Zoom Optical System
Short focal end $d\theta_{ab}$	119.63	36.88
Long focal end $d\theta_{ab}$	36.11	16.07
$d\theta_{RMS}$	69.68	25.04

## Data Availability

Data is contained within the article.
